# A cognitive–behavioural therapy programme for managing depression and anxiety in long-term physical health conditions: mixed-methods real-world evaluation of the COMPASS programme

**DOI:** 10.1192/bjo.2023.519

**Published:** 2023-08-11

**Authors:** Natasha Seaton, Rona Moss-Morris, Katrin Hulme, Hannah Macaulay, Joanna Hudson

**Affiliations:** Institute of Psychology, Psychiatry and Neuroscience, King's College London, UK

**Keywords:** Anxiety or fear-related disorders, cognitive–behavioural therapies, comorbidity, depressive disorders, qualitative research

## Abstract

**Background:**

Mental health comorbidities are common in physical long-term health conditions.

**Aims:**

We evaluate the effectiveness of COMPASS, a therapist-supported, digital cognitive–behavioural therapy programme specifically designed to treat anxiety/depression in the context of long-term conditions. We also investigate patient experiences of the programme.

**Method:**

We utilised a mixed-methods, non-randomised design. We analysed pre–post data from 76 patients with long-term conditions who were receiving psychological treatment (COMPASS) via local NHS services, using paired sample *t*-tests and Cohen's *d*, with depression, anxiety, distress and functional impairment self-report scales. Qualitative interviews explored patients’ experiences of using COMPASS. Twenty-one semi-structured interviews were completed and underwent inductive thematic analysis.

**Results:**

Patients who received COMPASS had significantly reduced depression (−2.47, 95% CI −3.7 to −1.3, P < 0.001; Cohen's *d* = −0.376), anxiety (−2.30, 95% CI −3.6 to −1.2, P < 0.001; Cohen's *d* = −0.420) and psychological distress (−4.87, 95% CI −7.0 to −2.7, P < 0.001; Cohen's *d* = −0.422) and significantly improved functional impairment (−3.00, 95% CI −4.8 to −1.2, P ≤ 0.001; Cohen's *d* = −0.282). Effect sizes were larger when analyses included only patients with clinically significant baseline symptoms: depression (−4.02, 95% CI −5.6 to −2.5, P < 0.001; Cohen's *d* = −0.701), anxiety (−3.60, 95% CI −5.3 to −1.9, P < 0.001; Cohen's *d* = −0.739), psychological distress (−5.58, 95% CI −7.9 to −3.2, P < 0.001; Cohen's *d* = −0.523), functional impairment (−3.28, 95% CI −5.4 to −1.1, P ≤ 0.001; Cohen's *d* = −0.355). Qualitative analysis yielded two meta-themes: engagement and integration of mental and physical health.

**Conclusions:**

Results suggest that COMPASS is effective in NHS settings, and is acceptable to patients. Content tailored to long-term conditions, therapist support and clear delivery strategies should be prioritised to aid intervention implementation.

Long-term conditions (LTCs) are chronic physical health conditions managed throughout life, for which there is no cure,^1^ such as coronary heart disease, diabetes and chronic obstructive pulmonary disease. Patients with LTCs face many challenges, including ongoing symptom management, challenging treatments and illness uncertainty.^2^ They experience higher rates of anxiety and depression compared with non-LTC populations, with 30% of patients with LTCs demonstrating clinically relevant symptoms.^3^ Comorbid depression and anxiety have negative consequences on LTC outcomes, including poorer prognosis, increased risk of mortality^4,5^ and 45–75% higher healthcare costs.^6^

Improving Access to Psychological Therapy (IAPT) services, now named NHS Talking Therapies, were set up in 2008 by NHS England to provide evidence-based, National Institute for Health and Care Excellence (NICE)-recommended psychological therapies to treat people with anxiety and depression in primary care. Subsequently, IAPT expanded its remit to include treatment of anxiety and/or depression in patients with LTCs, in line with the ‘UK NHS Five Year Forward View’ in 2016 specifying that patients with LTCs should receive integrated mental and physical healthcare.^7^ IAPT services keep outcome data on all patients coming through the service, facilitating a macro-level analysis of performance.^8^ Although IAPT published new LTC guidelines (IAPT-LTC) and expanded on its existing workforce, an analysis of real-world IAPT data demonstrates that patients with LTCs experience poorer mental health outcomes compared with their non-LTC counterparts.^9^ This deficit may be because of the use of conventional cognitive–behavioural therapy (CBT) protocols,^10^ which fail to address coping with symptoms, disease management and illness-specific concerns.^11,12^ Although there are some LTC-specific protocols (e.g. for diabetes and multiple sclerosis) that demonstrate better clinical outcomes, acceptability and engagement,^11–13^ IAPT services treat a wide range of physical LTCs and so either need a protocol for each condition or an approach that works across LTCs. To address this issue, our team developed the transdiagnostic model of adjustment to LTCs,^14^ to inform a broader approach to treating depression and anxiety in LTCs. We then designed a digital CBT programme, ‘COMPASS: Navigating your long-term condition’, around this model. Digital CBT standardises treatment protocols and, with minimal guided therapist support, achieves similar results as face-to-face therapy.^15^

COMPASS was developed using the UK Medical Research Council complex intervention guidance^16^ and IAPT-LTC guidelines set by NICE.^17^ The transdiagnostic model of adjustment to LTCs was used as an initial logic model and framework for mapping key intervention components. To address acceptability and usability issues often reported with digital interventions,^16^ the research team drew on the person-based approach.^18^ Following extensive engagement with patients and therapists, COMPASS was developed to focus on mechanisms known to trigger or maintain psychological distress in LTCs.^14^ In line with IAPT treatment pathways, COMPASS is supported by a trained therapist, delivering five or six 30 min fortnightly remote appointments. As part of the Medical Research Council guidance for developing and evaluating complex interventions,^16^ we sought to comprehensively evaluate the real-world implementation. Typically, it takes 17 years from development of psychological interventions to being implemented in routine care,^19^ potentially because behavioural randomised controlled trials (RCTs) are designed to show efficacy, often with little consideration for subsequent implementation.

## Study aims

The aim of this study was to assess the preliminary effectiveness and acceptability of COMPASS in a real-world setting, and to use the data to make recommendations for enhancements needed to the programme and/or treatment pathway before launching an efficacy RCT. A mixed-methods approach was used. The quantitative investigation aimed to use routinely collected data to assess the potential effectiveness of COMPASS (change in anxiety, depression, distress and functional impairment) delivered to patients with LTCs in routine National Health Service (NHS) care. In this study, the primary outcome was the combined Patient Health Questionnaire Anxiety and Depression Scale (PHQ-ADS) score, in line with studies that have found significant overlap between these measures in LTC populations.^20,21^ The qualitative investigation aimed to explore experiences and perceptions of patients enrolled in the programme, and provide guidance to improve the programme and its implementation in the future, to evaluate the acceptability of COMPASS.

## Method

The authors assert that all procedures contributing to this work comply with the ethical standards of the relevant national and institutional committees on human experimentation and with the Helsinki Declaration of 1975, as revised in 2008. This human study was approved by London NHS Quality Improvement Board (signed off by Director of Nursing at the relevant hospital Trust on 7 January 2019). No ethical approval was therefore required, in line with other quality improvement projects.^9^ This project utilised a pre–post cohort design with nested qualitative analysis. For the quantitative study, healthcare professionals documented participants’ verbal consent for anonymous data analysis. For the qualitative study, participants gave written informed consent to a researcher.

### Settings and patients

The project was set up at several locations. The key site was a large IAPT service (primary care), located in a multicultural area of London that ranks within the top 50 most deprived areas out of the 317 authorities in England.^22^ Patients can self-refer or be referred by a healthcare professional. In the IAPT setting, therapist support was provided by psychological well-being practitioners (PWPs) who have completed a 1-year training course in the delivery of psychological interventions. COMPASS was also launched in five secondary care services: two sites in July 2019 (xeroderma pigmentosum and neurofibromatosis), one site in October 2019 (oral medicine), one site in February 2020 (gastroenterology) and a further site in July 2020 (psychological medicine). In secondary care services, patients are typically referred to clinical psychologists by the medical consultants if mental health issues are raised during consultations.

### Service eligibility criteria

The following inclusion criteria were provided to the healthcare services to determine patients’ eligibility for COMPASS: age 18 years or over; speak English to a sufficiently high standard to allow them to interact with COMPASS; have an email address and internet access to allow them to register with COMPASS; and indication that depression/anxiety/distress is related their LTC, therefore being appropriate for an LTC pathway. Therapists were trained to assess this with specific prompts asking about whether patients’ mood was related to their LTC.

As part of national service guidelines, IAPT also require patients to have clinically relevant depression (a score of ≥10 on the Patient Health Questionnaire; PHQ-9^23^) or anxiety (a score of ≥8 on the Generalised Anxiety Disorder-7; GAD-7^24^) for inclusion. However, patients below these cut-offs can be eligible if they demonstrate a clear need for the service during clinical assessment. Valid reasons included within the service protocol were feeling that the questionnaires misrepresent how they feel, demonstrating functional impairment, requesting relapse prevention treatment or reporting long-term depression and/or anxiety symptoms despite demonstrating subclinical symptoms within the questionnaire timeframe (both the PHQ-9 and GAD-7 ask about the past 2 weeks). In line with service protocols for online CBT programmes and other low-intensity interventions, patients were deemed ineligible if they had a cognitive impairment, active suicidal plans, bipolar depression or schizophrenia.

### Study eligibility criteria

For the study, inclusion criteria were receipt of COMPASS as the primary intervention and diagnosis of an LTC. Patients were excluded if they had a persistent physical symptom (PPS) diagnosis only, such as irritable bowel syndrome or fibromyalgia. This was because COMPASS specifically treats anxiety and depression in LTCs, and different protocols are recommended for PPS.^25,26^

Additionally, patients were excluded from the quantitative analysis if they had only completed one set of outcome measures, as pre–post scores could not be calculated. In the qualitative analysis, patients were eligible if they were enrolled in the COMPASS programme as part of the IAPT or hospital services, from the initial embedding phase in July 2019 to 30 June 2021. This included patients that were non-engagers, defined as not completing two outcome measures on the programme.

### COMPASS intervention

COMPASS is a therapist-supported, 11-session, digital CBT programme. Recommended therapist support is five to six 30 min fortnightly calls, over a period of 10–12 weeks. In IAPT, patients were referred to COMPASS after they completed their assessment telephone call, were deemed eligible for COMPASS and selected COMPASS as their preferred treatment option. In the hospital-based specialist services, clinicians referred patients they considered to be distressed to the clinical psychologist, who assessed the patient against the COMPASS inclusion/exclusion criteria.

Once referred to COMPASS, a patient receives an automated welcome email including their COMPASS registration details. Therapists send an in-site message to patients, explaining the support process, scheduling the first telephone call and encouraging them to complete the first introductory module. If a patient has not registered with the programme after 2 weeks, an ‘on-boarding’ technical support call is provided to assist patients who are experiencing difficulties accessing the programme.

### Therapist training

In the IAPT service, during the implementation period, COMPASS was delivered by ten PWPs who received training in providing support with COMPASS. PWPs deliver low-intensity treatments in IAPT, such as online CBT, guided self-help and psychoeducation groups, and are trained to assess and support people with common mental health problems, such as mild-to-moderate anxiety and/or depression.^27^ Conversely, in the five hospital-based specialist LTC services, clinical psychologists are responsible for providing therapy as part of integrated multidisciplinary care. A clinical psychologist was trained to deliver COMPASS in each hospital service; one service (gastroenterology) additionally trained an assistant psychologist.

### COMPASS outcome monitoring system

COMPASS has capability to collect and record clinical outcomes for patients. Immediately after registration with the programme, patients complete baseline measures of the PHQ-9,^23^ GAD-7^24^ and Work and Social Adjustment Scale (WSAS)^28^ (described below). These questionnaires are automatically and routinely sent by the COMPASS programme at 48 h before a therapist-scheduled appointment and at discharge.

All questionnaire data is stored online on the COMPASS system. Pseudo-anonymised data reports were downloaded from the COMPASS system on 30 June 2021, 18 months after the initial implementation period in the IAPT service was completed and 24 months after implementation in the specialist acute services.

### Measures of clinical outcome

Clinical outcomes for this study were based on three questionnaires that IAPT routinely collects before each appointment and at discharge.

- Depression was measured with the PHQ-9.^23^ The PHQ-9 is routinely used in clinical and research settings, with meta-analytic evidence of validity, reliability and diagnostic capability.^29^ The self-report questionnaire contains nine items, where each item corresponds to a diagnostic DSM criterion for depression.^30^ Each item is rated on a four-point scale, with aggregated scores ranging from 0 to 27. A score ≥10 indicates clinically significant depression.^31^ At all study time points, Cronbach's alpha was 0.90.
- Anxiety was measured with the GAD-7.^24^ The GAD-7 is reliable and valid in clinical populations.^24^ It has seven self-report items, rated on a four-point scale. Responses are accumulated and range from 0 to 21, with a score ≥8 indicating clinically significant anxiety.^31^ At all study time points, Cronbach's alpha was 0.91.
- Psychological distress was measured according to the PHQ-ADS,^21^ which is a combined PHQ-9^23^ and GAD-7^24^ score. The PHQ-ADS is thought to be more appropriate for patients with LTCs, given the cooccurrence of anxiety and depression in LTCs.^21^ The PHQ-ADS has shown structural validity in patients with LTCs,^20,21^ with a score ≥10 indicating clinically significant levels of psychological distress.^21^ At all study time points, Cronbach's alpha was 0.94.
- Functional impairment was measured with the WSAS.^28^ It includes five self-report items, each referring to an aspect of life that may be impaired. Each item is rated on a nine-point scale (total scores range from 0 to 40), with a score ≥10 indicating caseness.^28^ The WSAS has good reliability and validity. The Cronbach's alpha ranged from 0.88 (baseline) to 0.94 (follow-up).

### Data gathering and analysis

#### Statistical analyses

Following a 5-month pre-implementation phase, where workflows and implementation issues were negotiated and settled (July 2019 to December 2019), the services were using the programme in accordance with implementation guidelines by January 2020. The quantitative data extraction phase ran from 1 January 2020 to 30 June 2021. Descriptive statistics were computed to summarise the demographic data. Questionnaire totals were computed in accordance with guidelines stipulated by the authors,^23,24,28^ and in accordance with routine IAPT service analyses.^31^

Paired sample *t*-tests were used to assessed differences between pre- and post-intervention scores. A sensitivity analysis was performed to investigate patients who had greater severity of symptoms at baseline according to the criteria used in IAPT services (scores of ≥10 for the PHQ-9 and ≥8 for the GAD-7),^31^ in addition to the criteria applied for this study (scores of ≥10 for the PHQ-ADS^20,21^ and ≥10 for the WSAS^28^). For each outcome measure, mean changes in questionnaire scores, *t*-statistics, *P*-values, Cohen's *d* effect sizes, s.e. and 95% confidence intervals were computed. A 5% alpha level was applied for all statistical tests. Recovery, a key metric within IAPT, was also calculated and was defined as the percentage of patients scoring below clinical cut-off points on self-report measures of anxiety and depression after treatment, having been above cut-off points on either the PHQ-9 or GAD-7 at baseline.^31^ All data analysis was performed with Stata for Windows version 16.0 software.

#### Qualitative data gathering and analyses

Patients who had been enrolled and discharged from COMPASS (irrespective of engagement) and had consented to be contacted for interview were approached. The semi-structured interview schedule included open-ended questions and prompts to facilitate the exploration of patients’ experiences being referred to and using the intervention (Supplementary Material available at https://doi.org/10.1192/bjo.2023.519). Telephone interviews were conducted by one researcher (N.S.) between November 2019 and January 2021. Interviews were securely recorded, uploaded and transcribed; three by a researcher (N.S.) and 18 by a professional service. Interviews ranged in length from 14 to 57 min (mean: 32 min 9 s; s.d. 13 min 12 s).

Data were inductively analysed with thematic analysis techniques in accordance with Braun and Clarke's thematic analysis guidelines.^32^ Two members of the research team (H.M. and N.S.) separately familiarised themselves with the data and did line-by-line transcript coding. Choice codes were reviewed and examined between researchers, ensuring that concordance was met, enhancing intercoder reliability. Codes were grouped into potential themes, which were discussed within the research team before a final thematic map was constructed.

## Results

### Quantitative results

**Fig. 1:**
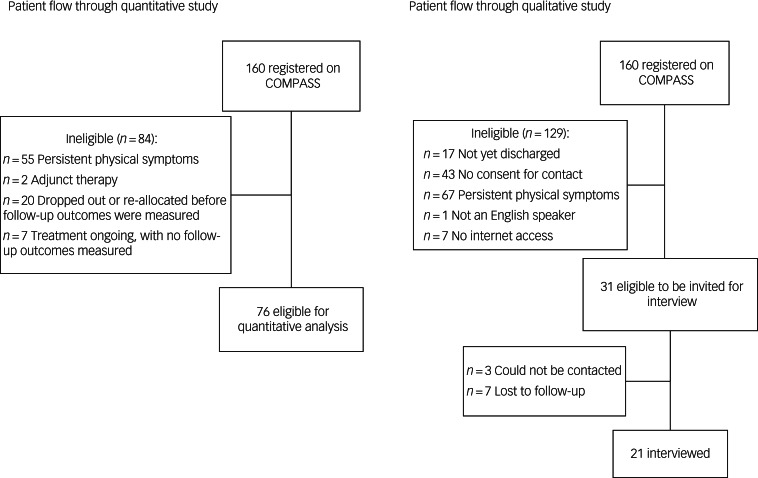
Flow of patients through quantitative and qualitative studies.

Of the 160 patients registered with COMPASS in the time period, 55 were excluded as they had a PPS diagnosis without an LTC diagnosis. Two were excluded as they were receiving COMPASS as an adjunct therapy, 20 were discharged before giving two scores (dropped out because of disengagement with the programme or a re-allocation to a more suitable treatment) and seven did not yet have two outcomes but had treatment ongoing. During the COMPASS registration process, all patients (*N* = 160) consented to anonymised data storage and analysis as part of the programme. Participant flow is shown in Fig. 1.

A total of 76 patients met eligibility criteria, with 59 patients from IAPT and 17 patients from the specialist acute services. Patients were mostly female (63.1%) and 46.1% were White, with an average age of 42.9 years (s.d. 12.2). All continuous variables were normally distributed. Table 1 summarises descriptive data for demographics and clinical variables.

**Table 1:** Demographic and clinical factors of the sample

	*n* or mean (s.d.)	%
Gender		
Female	48	63.1
Male	26	34.2
Other	2	2.6
Ethnicity		
Asian or Asian British	5	6.6
Black or Black British	21	27.6
Mixed ethnicity	3	4.0
White British	35	46.1
Other	1	1.3
Unknown	11	14.5
Age, years	42.9 (12.2)	
Depression		
Baseline depression	12.6 (6.7)	
At caseness for depression	46	60.5
Depression categories		
None	8	10.5
Mild	22	29.0
Moderate	18	23.7
Severe	28	36.8
Anxiety		
Baseline anxiety	10.4 (6.0)	
At caseness for anxiety	51	67.1
Anxiety categories		
None	13	17.1
Mild	29	38.2
Moderate	10	13.2
Severe	24	31.6
Functional impairment		
Baseline functional impairment	20.6 (10.4)	
At caseness for functional impairment	65	85.5
Psychological distress		
Baseline psychological distress	22.9 (11.7)	
At caseness for psychological distress	67	88.2
Number of completed sessions	4.0 (3.7)	
Time spent on the programme, min	153.3 (204.4)	
Site		
Gastroenterology	6	7.9
IAPT	59	77.6
Neurofibromatosis	5	6.6
Oral medicine	1	1.3
Psychological medicine	1	1.3
Xeroderma pigmentosum	4	5.3
Most challenging LTC		
Diabetes	11	14.5
Inflammatory bowel disease	9	11.8
Arthritis	7	9.2
Asthma	6	7.9
Musculoskeletal disorder	5	6.6
Neurofibromatosis	5	6.6
Endometriosis	4	5.3
Xeroderma pigmentosum	4	5.3
Lupus	3	3.9
Multiple sclerosis	3	3.9
Cancer	2	2.6
Neurological disorder	2	2.6
Thyroid condition	2	2.6
Other	13	17.1

IAPT, Improving Access to Psychological Therapies; LTC, long-term condition.

Table 2 shows the pre–post analysis for the total sample. PHQ-ADS scores changed by −4.87 points (*P* < 0.001), yielding a medium effect size (Cohen's *d* = −0.42). There were also small-to-medium significant changes for depression (mean −2.76, *P* < 0.001, Cohen's *d* = −0.38), anxiety (mean −2.30, *P* < 0.001, Cohen's *d* = −0.42) and functional impairment (mean −3.00, *P* < 0.001, Cohen's *d* = −0.28). Larger mean differences across outcomes were observed when the sensitivity analysis investigated individuals who had clinically significant symptoms at baseline according to *a priori* defined cut-offs. Of the 76 patients, the number who were above the clinical threshold was 46 (60.5%) for depression, 51 (67.1%) for anxiety and 67 (88.2%) for distress, with 65 (85.5%) indicating significant functional impairment. Effect sizes were large for depression and anxiety (depression: mean −4.02, Cohen's *d* = −0.73, *P* < 0.001; anxiety: mean −3.60, Cohen's *d* = −0.58 *P* < 0.001), medium for psychological distress (mean −5.58, Cohen's *d* = −0.52, *P* < 0.001) and small-to-medium for functional impairment (mean −3.28, Cohen's *d* = −0.36, *P* < 0.001).

**Table 2:** Changes in outcome measures from baseline to most recent outcome measure

	Group	*n*	Mean	s.e.	Confidence interval		*t*-Test	*P*-value	Cohen's *d*
					Lower bound	Upper bound			
Change in PHQ-9 score	All patients	76	−2.47	0.60	−3.7	−1.3	−4.154	0.0001***	−0.376
	Not at caseness (<10)	30	−0.10	0.74	−1.6	1.4	−0.134	0.4471	−0.029
	At caseness (≥10)	46	−4.02	0.78	−5.6	−2.5	−5.157	<0.0001***	−0.701
Change in GAD-7 score	All patients	76	−2.30	0.62	−3.6	−1.2	−3.841	0.0003***	−0.420
	Not at caseness (<8)	25	0.08	0.65	−1.3	1.4	0.124	0.5488	0.028
	At caseness (≥8)	51	−3.60	0.83	−5.3	−1.9	−4.370	0.0001***	−0.739
Change in WSAS score	All patients	76	−3.00	1.24	−4.8	−1.2	−3.322	0.0014**	−0.282
	No significant functional impairment (<10)	11	−1.36	0.69	−2.9	.2	−1.973	0.0384*	−0.485
	Significant functional impairment (≥10)	65	−3.28	1.05	−5.4	−1.1	−3.129	0.0013**	−0.355
Change in PHQ-ADS	All patients	76	−4.87	1.09	−7.0	−2.7	−4.460	<0.0001***	−0.422
	Not at caseness (<10)	9	0.44	2.02	−4.2	5.1	0.219	0.5840	0.086
	At caseness (≥10)	67	−5.58	1.18	−7.9	−3.2	−4.711	<0.0001***	−0.523

PHQ-9, Patient Health Questionnaire-9; GAD-7, Generalised Anxiety Disorder-7; WSAS, Work and Social Adjustment Scale; PHQ-ADS, Patient Health Questionnaire Anxiety and Depression Scale.

*P≤0.05, **P≤0.01, ***P≤0.001.

Examining treatment outcomes according to IAPT recovery metrics (see Method section), 56 out of 76 (73.7%) patients were above clinical cut-offs on either anxiety or depression at baseline. Twenty-five (44.6%) were classified as recovered post-treatment (scores were below their clinical cut-offs).

### Qualitative results

#### Flow of recruitment for qualitative study

By 30 June 2021, 143 participants had been discharged from COMPASS, of whom 100 (69.9%) had consented to be contacted for interview. A total of 69 patients were ineligible for interview: 67 had only a PPS diagnosis, one did not speak English and one had no internet or smart device access. Of the 31 eligible participants, three could not be contacted at all and seven were lost to follow-up (see Fig. 1). This gave an interview consent rate of 67.7%.

Interviewing ceased when the research team agreed sufficient depth of understanding had been acquired in relation to emergent codes and themes.^33^ A total of 21 COMPASS patients were interviewed. The average age was 43.0 (s.d. 16.4) years and 66.7% were female. Eighteen LTC diagnoses were recorded, with six participants having at least one comorbidity. Table 3 shows full demographic and clinical data.

**Table 3:** Demographic and diagnosis data for qualitative interview participants

	*n* (%) or mean (s.d.)
Gender	
Female	14 (66.7%)
Male	7 (33.3%)
Ethnicity	
Asian or Asian British	2 (9.5%)
Black or Black British	8 (38.1%)
Mixed, White and Black	1 (4.8%)
White or White British	9 (42.9%)
Unknown	1 (4.8%)
Age, years	43.0 (16.4)
Long-term condition	
Diabetes	5 (23.8%)
High blood pressure	4 (19%)
Arthritis	3 (14.3%)
Polycystic ovary syndrome	2 (9.5%)
Asthma	2 (9.5%)
Thyroid problem	2 (9.5%)
Migraine	2 (9.5%)
Inflammatory bowel disease	1 (4.8%)
Graves’ disease	1 (4.8%)
Epilepsy	1 (4.8%)
Multiple sclerosis	1 (4.8%)
Lupus	1 (4.8%)
Skin condition	1 (4.8%)
Chronic pain, including fibromyalgia	1 (4.8%)
Respiratory tract condition	1 (4.8%)
Xeroderma pigmentosum	1 (4.8%)
Coronary heart disease	1 (4.8%)
Sarcoidosis	1 (4.8%)

Table 4 summarises the two meta-themes derived from the inductive thematic analysis: engagement and integration of mental and physical health. These themes highlight patients’ experience of using COMPASS and barriers and facilitators affecting uptake and utilisation.

**Table 4:** Meta-themes, themes and subthemes identified through inductive thematic analysis

Meta-theme	Theme	
Engagement: factors that appear to impact, enhance and prevent engagement	The advantages and disadvantages of online therapy: attitudes and beliefs regarding advantages and disadvantages of digital therapy	
	Interactions with the service: confusion among patients negatively affecting engagement	
	Human connection: necessary and important feature of COMPASS	
Integration of mental and physical health: relationship between facets of health and well-being within the programme and appraisal of LTC-tailored content	COMPASS content and coping with my LTC: perception and experiences of LTC-tailored content on COMPASS	Subthemes:
		LTC management
		Behaviour change
		LTC outlook
		Mood and emotional control
		Social connectedness
	Life experiences: the bidirectional impact of mental and physical health for patients with LTCs in their daily life	

LTC, long-term condition.

#### Meta-theme 1: engagement

The meta-theme of engagement subsumed three themes: the advantages and disadvantages of online therapy, interactions with the service and human connection.

##### The advantages and disadvantages of online therapy

Participants had a range of positive, negative and mixed reactions toward online therapy. Positive opinions included accessibility, personalisation, availability, convenience, flexibility with scheduling, lower pressure, quicker to get an appointment and reduced social anxiety.

‘You can work with your own timings and that seemed to me, to be a very good option. Because I'm working from home, working in an office, it's nice to do this stuff when you have peace and quiet, when you've got an hour, two hours, to sit down and be with your thoughts.’ Participant 012

Some participants perceived the workload of online treatment to be large, citing its incompatibility with their mental or physical health status.

‘When I joined, I was quite physically unwell, I found it difficult to engage … I think having to physically sit there and try to engage with it, was sometimes difficult. If you're feeling quite low, I think it's quite hard because it's daunting.’ Participant 001

People who were negative about online treatments often mentioned low technological literacy and/or a preference for face-to-face therapy. These views were often accompanied by scepticism that an online treatment would work.

‘I'm very not good at computers …. You know when you're not good at something, you always panic before you do it. I'm already panicked when I go to the computer.’ Participant 010

‘I wanted real counselling, and I preferred that kind of personal communication. This felt exactly the opposite, very impersonal. Having to concentrate, I don't really see how it could be helpful … It's a no from me, I do not like online programme.’ Participant 015

##### Interactions with the service

Participants discussed a range of communication issues, including confusion with triage, lack of clarity with appointment scheduling and discharge, and incorrect or insufficient explanation of the COMPASS programme and the therapist support. These reasons were often a barrier to engagement.

‘I phoned them eventually. [The service] said I was [discharged] so I had to go back, but I didn't know where to start over again. I've not had anything since then. I'm just waiting to see what happens … I'm not getting information.’ Participant 003

Moreover, despite choice of therapy being part of routine care some participants felt COMPASS was not their choice, or that it was a waitlist option rather than an actual treatment. This tended to coincide with scepticism and negative expectations of the programme.

‘It's a bit of a challenge for the patient to say why this is going to be a benefit to them because they didn't choose it in the first place.’ Participant 018

##### Human connection

Most participants positively appraised the presence of the therapist, with a subsection saying that the therapist was essential for engagement. They appreciated that therapists provided emotional support, additional insight, perspectives and information tailored to their needs; encouragement to log on; accountability for progress and technical support and guidance.

‘It was like they hadn't forgotten about you and someone being at the end of the line. I found that encouraging.’ Participant 014

‘[The therapist] adds another level to the information that is already on COMPASS because the calls make it tailored towards my answers to the questions and discussing the answers and how that impacted me helped me move forward.’ Participant 006

Moreover, participants found that the ‘patient stories’ provided hope, solidarity and motivation by providing achievable solutions to relevant and relatable circumstances.

‘[The patient stories put] things into a different perspective … just the idea that somebody else has had similar thoughts or experiences [and] that they've found solutions which is encouraging.’ Participant 009

#### Meta-theme 2: integration of mental and physical health

Integration of mental and physical health is comprised of two themes: ‘COMPASS content and coping with my LTC’ and participants’ life experiences.

##### COMPASS content and coping with my LTC

Participants positively appraised the LTC-tailored content, falling into five subthemes (see Table 4). Within LTC management, participants spoke about better symptom and disease management. The content about stress and physical health relationship was appreciated, especially as they cited that this was overlooked by themselves or healthcare professionals.

‘I felt like there was quite a lot of essentially CBT in some of COMPASS, then on top of that [you have] the lifestyle, the stress component, the physical health … I'd say I'm getting more from COMPASS than I did from the talking therapies that I've had previously.’ Participant 021

In the subtheme ‘behaviour change’, problem-focused coping aspects of the programme were positively appraised, helping participants establish better health behaviours (sleep, diet, exercise, mindfulness), which in turn helped symptom management and mood.

‘[COMPASS] motivated me to do [exercise and diet changes …. It was a way for me to de-stress but also to feel good about myself … I never realised before how much my physical health was affecting my mental health …. [improving my sleep and diet] had a positive impact on my mental health.’ Participant 006

Following COMPASS, participants’ LTC outlook improved. They described having more acceptance of their LTC, understanding their diagnosis and identity better, and increasing resilience to LTC challenges.

‘I think it's really helped with my ability to view my illnesses and manage my depression, because I've stopped taking anti-depressants now … instead of just the doom and gloom that I initially thought with my diagnosis, it made me think about potentially other things out there.’ Participant 006

Participants had improved mood and emotional control, as the sessions helped with challenging unhelpful thoughts, managing mood and processing emotions. Some participants mentioned that these techniques helped with relaxation, improving feelings of depression and loneliness and employing a positive mentality.

‘[The relaxation and mindfulness] helped me to put the worry aside and to think just about what I was doing there, rather than worrying about what was happening in the pain. [it] helped me relax and sleep better, even though the pain was still there.’ Participant 013

Participants mentioned improved social connectedness following therapy. COMPASS helped them navigate social situations and ‘speak to people and get the support that I need’ (Participant 006) from healthcare providers, friends and family.

##### Life experiences

Participants described how mental health concerns were related to psychological adjustment to LTCs. Symptoms negatively affected mental health and imposed limits on daily activity; concurrently, poor mental health often led to negative health behaviours or avoidance. Participants expressed that illness uncertainty and illness-related stigma induced worries, low mood and difficulties in work and social situations.

‘I work in a small school, and I felt that asking for reasonable adjustment was burdensome. I felt as though I was an additional expense to my employer, and this was made worse by the fact that we are a small school.’ Participant 005

## Discussion

The study aimed to assess the potential real-world effectiveness and acceptability of COMPASS, a therapist-supported online CBT programme for treating anxiety and depression in patients with LTCs. Patients with LTCs receiving the COMPASS intervention in NHS services showed significant improvements in psychological distress, depression, anxiety and functional impairment. The effect sizes were larger and remained statistically significant when only patients with clinically significant scores at baseline were analysed.

The qualitative investigation yielded two meta-themes regarding patient experiences and attitudes regarding the programme: engagement and integration of mental and physical health. Online therapy was perceived to confer advantages with regards to access and scheduling; however, some patients struggled with technological aspects, where others felt their mental and/or physical health were too severe for them to engage. Some interactions with the service presented major barriers to engagement, but the presence of a therapist increased motivation and facilitated engagement in the programme. Human connection was highly valued, provided by both the therapist support and the relatable, relevant ‘patient stories’ in the programme. Patients positively appraised the LTC-tailored content, responding well to the integration of mental health and physical health within COMPASS, an overlap that was clearly reflected in personal accounts of having an LTC.

Past non-tailored CBT interventions for patients with LTCs have reported small effect sizes.^9,10^ The current study suggests COMPASS, when delivered in a real-world setting, shows medium effect sizes for improvements in psychological distress, depression and anxiety, considered to constitute clinically meaningful change,^21^ as well as a small effect for functional impairment. The qualitative findings that patients positively appraised relevant patient examples and LTC-specific content provide some support for the transdiagnostic model of psychological adjustment to LTCs,^14^ and may underly the promising quantitative results. Patients with LTCs often report dissatisfaction in their mental healthcare including finding primary mental health disorder materials irrelevant.^12^ Therapists also acknowledge the need for LTC-tailored treatments.^34^ Tailored content may promote engagement and accordingly improve outcomes. Patients were included in every iteration of COMPASS development, which likely enhanced the relevance of COMPASS and underlines the importance of the person-based approach^18^ in developing interventions. This study's findings were used to make additional improvements to COMPASS and the therapist training before conducting an RCT. The RCT protocol is published^35^ and will be reported elsewhere.

The qualitative findings indicate the preference and, for some patients, necessity of therapist presence for digital therapies. This concurs with past research, where human support is key to acceptability, usability and effectiveness of digital health interventions;^36^ conversely, patients receiving non-guided interventions feel unsupported and demonstrate low adherence.^37,38^ Guided interventions appear more effective at improving depression and anxiety,^39^ as therapists are likely to increase motivation and, accordingly, improve adherence.^40^

Within the interviews, there were contradictory findings regarding the acceptability of online treatment. Participants finding remote therapy convenient is heavily supported by the literature;^41^ however, a meta-synthesis investigating digital therapy for adults with depression, anxiety and/or somatoform disorders has, like ours, found that some patients feel they require face-to-face contact to engage in psychological treatment.^36^ Given the diversity of lived experience, it is unsurprising that online interventions elude unanimous appeal. Indeed, our finding that lower technological literacy hindered motivation and engagement is corroborated by systematic review evidence, highlighting that technological literacy is essential to acceptability of digital therapies.^42^ Although the emergence of digital interventions is an exciting opportunity to provide high-quality treatments to large populations, considerations must be given to populations with lower technological literacy. In NHS settings, to guarantee that care is appropriate for the individual, practitioners should ensure a patient has internet access and confidence using a smartphone/computer before referring patients to online therapy programmes.

Additionally, patients experiencing debilitating physical and/or psychological symptoms found it difficult to engage. As this finding developed, the research team streamlined programme content to help reduce patient burden. However, the needs of these patients should be accommodated, perhaps through blended therapy, whereby face-to-face treatments are supplemented with online CBT or other high-intensity treatment alternatives. Such protocols would compare with treatments recommended for patients with severe mental health difficulties, chronic pain and chronic fatigue in IAPT services.^17^

Finally, disconnects in communication with the healthcare service were a barrier to engagement, including incorrect and insufficient information about treatment options. Importantly, these interviews occurred during the COVID-19 pandemic, when mental healthcare services were under immense pressure. Nonetheless, this finding suggests that how an intervention is presented to patients and normalised within a service is key to patients’ acceptance of the therapy.

### Strengths and limitations

This study was naturalistic, thus providing direct insight into the acceptability and effectiveness of the COMPASS programme in a real-world NHS setting, including patients that may normally be underrepresented in conventional research projects. Despite being ethnically diverse, the study sample was small. Moreover, as this was a quality improvement project recruitment ineligibility data could not be fully captured and patients who declined COMPASS could not be interviewed. Therefore, a hybrid implementation RCT^43^ with a larger sample size would assess the effectiveness of COMPASS alongside its real-world implementation.

In conclusion, COMPASS is an effective treatment for patients with LTCs who engage with the programme in real-world NHS settings. Participants comment specifically on the value of therapist support and LTC-tailored treatment. Given this, COMPASS may help to improve the poorer outcomes among patients with LTCs who access IAPT services.^9^ However, the qualitative findings highlight two core factors that need addressing to improve the acceptability of COMPASS. First, technical literacy was a barrier to engagement for some. Developing an app version of COMPASS as opposed to a web version may tackle this conflict. However, IAPT and specialist healthcare services offer treatments in multiple modalities, and future research could explore how to better match treatments to the most appropriate mode of delivery at the assessment and triage stage of therapy. Second, insufficient information and confused communication from the service negatively affected treatment expectations. When embedding treatments like COMPASS into services, continuous audit and feedback cycles would help to identify when the COMPASS treatment protocol is not being delivered as intended, and provide staff with opportunities to discuss their barriers to delivery.

## Supporting information

Seaton et al. supplementary material Seaton et al. supplementary material

## Data Availability

The data used in the study are available on request from the corresponding author, J.H. The data are not publicly available they could compromise the privacy of the participants.
