# Supplementary material for: A cognitive–behavioural therapy programme for managing depression and anxiety in long-term physical health conditions: mixed-methods real-world evaluation of the COMPASS programme
Source: BJPsych Open. 2023 Aug 11;9(5):e153. doi: 10.1192/bjo.2023.519 (PMC10594095; doi:10.1192/bjo.2023.519)
Supplement: Seaton et al. supplementary material [file S2056472423005197sup001.docx]

**Patient Interview Schedule**

**[ENGAGERES]**

I’m contacting a number of people who have used COMPASS to find out about their experiences of the online programme and the support of your guide.

**[NON-ENGAGERS or NON-REGISTERED]**

I’m contacting a number of people who were referred to COMPASS from [SERVICE] to find out about their thoughts towards online programmes for managing depression and anxiety in LTCs and people’s experience of [SERVICE].

**[ALL]**

Anything you can tell me about your experiences including good and bad points would be useful. Although I am working within [SERVICE], I am an independent researcher, so please feel free to be completely honest about your satisfaction with the health services and the online programme. Do you have any questions before we start? Are you happy to continue?

**Section A. Assessment**

**[ALL PARTICIPANTS]**

1. How did you hear about IAPT?
2. Tell me about your experience of the assessment process?
3. Is there anything that could have been done differently?
4. How would you like health care professionals to talk about your emotions?
5. How was COMPASS explained to you when you had your first assessment at **[SERVICE]?**
6. What were your reasons behind choosing COMPASS as a treatment option?

**Section B. Experience of COMPASS**

What was your experience of COMPASS?

**[ENGAGERS]**

Additional prompts to be used flexibly if necessary:

1. What were your expectations of COMPASS before trying it?
2. How did you find COMPASS treatment after a while of using it?
3. How did you use COMPASS?
4. What did you like about COMPASS?
5. What did you dislike about COMPASS?
6. How have your feelings of distress been since you’ve been using COMPASS?
7. How do you feel you have been coping with your LTC since using COMPASS?
8. Can you tell me about anything that you feel changed whilst receiving COMPASS? (Symptoms, Thoughts, Feelings, Lifestyle, Social/Relationships)
9. How does Compass compare to other forms of treatment?
10. Can you tell me about how your guide supported you through COMPASS
    1. Can you explain how you felt about the guide?
    2. Do you feel as if the guide understood your challenges?
11. Looking back on this treatment now....
    1. What do you think about this treatment for managing your emotional distress?
    2. How do you feel about the use of this to manage emotional distress for you and others in the future?
    3. Was it useful and helpful to you?
    4. Was the information included relevant to your needs?
    5. Is there anything more you would have liked?
12. When you were discharged from the programme, you should have received an email reminder to log back in and do the final outcome questionnaires. Do you remember receiving such an email?
13. Was it helpful to have physical health and mental health together?
14. Did you feel as though COMPASS was suitable to someone like you in terms of your age, gender, ethnicity, disability status?
    1. Any aspects in particular?
    2. What could be done to improve this?

**[NON-ENGAGERS]**

Additional prompts to be used flexibly if necessary:

1. What reasons were there that meant you did not end up using COMPASS?
2. Do you think an online platform would help you with your LTC and related distress? (*coherence – NPT*)
3. How would you like an online platform to support you?
4. Would message or telephone support from a guide/therapist be useful?

**[NON-REGISTERED]**

Additional prompts to be used flexibly if necessary:

1. What reasons were there that meant you did not end up signing up to COMPASS?
2. Do you think an online platform would help you with your LTC and related distress?
3. How would you like an online platform to support you?
4. Would message or telephone support from a guide/therapist be useful?

**Section C. Support**

**[ENGAGERS]**

Can you tell me about how found your sessions with the guide?

1. How were you supported using COMPASS?
   1. *(probe if necessary)* what did you think of the therapist contact?
   2. Role of therapist / route to triage / interactions with therapist
2. Was there anything you felt the guide offered that the COMPASS online sessions on their own couldn’t offer?
3. What was helpful about the guide?
4. What was unhelpful?
5. How would you like to be supported when using COMPASS?
6. Do you think the guide is needed?
